# Regulated necrosis pathways: a potential target for ischemic stroke

**DOI:** 10.1093/burnst/tkad016

**Published:** 2023-11-18

**Authors:** Kaidi Ren, Jinyan Pei, Yuanyuan Guo, Yuxue Jiao, Han Xing, Yi Xie, Yang Yang, Qi Feng, Jing Yang

**Affiliations:** Department of Pharmacy, The First Affiliated Hospital of Zhengzhou University, No. 1 Jianshe Dong Road, ErQi District, Zhengzhou 450052, China; Henan Key Laboratory of Precision Clinical Pharmacy, Zhengzhou University, No. 1 Jianshe Dong Road, ErQi District, Zhengzhou 450052, China; Henan Engineering Research Center for Application & Translation of Precision Clinical Pharmacy, No. 1 Jianshe Dong Road, ErQi District, Zhengzhou University, Zhengzhou 450052, China; Quality Management Department, Henan No. 3 Provincial People’s Hospital, Henan No. 3 Provincial People’s Hospital, Zhengzhou 450052, China; Department of Pharmacy, The First Affiliated Hospital of Zhengzhou University, No. 1 Jianshe Dong Road, ErQi District, Zhengzhou 450052, China; Henan Key Laboratory of Precision Clinical Pharmacy, Zhengzhou University, No. 1 Jianshe Dong Road, ErQi District, Zhengzhou 450052, China; Henan Engineering Research Center for Application & Translation of Precision Clinical Pharmacy, No. 1 Jianshe Dong Road, ErQi District, Zhengzhou University, Zhengzhou 450052, China; Quality Management Department, Henan No. 3 Provincial People’s Hospital, Henan No. 3 Provincial People’s Hospital, Zhengzhou 450052, China; Department of Pharmacy, The First Affiliated Hospital of Zhengzhou University, No. 1 Jianshe Dong Road, ErQi District, Zhengzhou 450052, China; Henan Key Laboratory of Precision Clinical Pharmacy, Zhengzhou University, No. 1 Jianshe Dong Road, ErQi District, Zhengzhou 450052, China; Henan Engineering Research Center for Application & Translation of Precision Clinical Pharmacy, No. 1 Jianshe Dong Road, ErQi District, Zhengzhou University, Zhengzhou 450052, China; Department of Neurology, The First Affiliated Hospital of Zhengzhou University, No. 1 Jianshe Dong Road, ErQi District, Zhengzhou University, Zhengzhou 450052, China; Research Center for Clinical System Biology, Translational Medicine Center, No. 1 Jianshe Dong Road, ErQi District, The First Affiliated Hospital of Zhengzhou University, Zhengzhou 450052, China; Research Institute of Nephrology, The First Affiliated Hospital of Zhengzhou University, No. 1 Jianshe Dong Road, ErQi District, Zhengzhou 450052, China; Department of Integrated Traditional and Western Nephrology, The First Affiliated Hospital of Zhengzhou University, No. 1 Jianshe Dong Road, ErQi District, Zhengzhou 450052, China; Henan Province Research Center for Kidney Disease, The First Affiliated Hospital of Zhengzhou University, No. 1 Jianshe Dong Road, ErQi District, Zhengzhou 450052, China; Department of Pharmacy, The First Affiliated Hospital of Zhengzhou University, No. 1 Jianshe Dong Road, ErQi District, Zhengzhou 450052, China; Henan Key Laboratory of Precision Clinical Pharmacy, Zhengzhou University, No. 1 Jianshe Dong Road, ErQi District, Zhengzhou 450052, China; Henan Engineering Research Center for Application & Translation of Precision Clinical Pharmacy, No. 1 Jianshe Dong Road, ErQi District, Zhengzhou University, Zhengzhou 450052, China

**Keywords:** Ischemic stroke, Necroptosis, Pyroptosis, Ferroptosis, Pathanatos, mPTP-mediated necrosis, Oncosis

## Abstract

Globally, ischemic stroke causes millions of deaths per year. The outcomes of ischemic stroke are largely determined by the amount of ischemia-related and reperfusion-related neuronal death in the infarct region. In the infarct region, cell injuries follow either the regulated pathway involving precise signaling cascades, such as apoptosis and autophagy, or the nonregulated pathway, which is uncontrolled by any molecularly defined effector mechanisms such as necrosis. However, numerous studies have recently found that a certain type of necrosis can be regulated and potentially modified by drugs and is nonapoptotic; this type of necrosis is referred to as regulated necrosis. Depending on the signaling pathway, various elements of regulated necrosis contribute to the development of ischemic stroke, such as necroptosis, pyroptosis, ferroptosis, pathanatos, mitochondrial permeability transition pore-mediated necrosis and oncosis. In this review, we aim to summarize the underlying molecular mechanisms of regulated necrosis in ischemic stroke and explore the crosstalk and interplay among the diverse types of regulated necrosis. We believe that targeting these regulated necrosis pathways both pharmacologically and genetically in ischemia-induced neuronal death and protection could be an efficient strategy to increase neuronal survival and regeneration in ischemic stroke.

Highlights

- This review aims to provide a systematic view of the role of regulated necrosis in neurons, glia cells, platelets and brain microvascular cells in response to ischemia stress.
- We also review recent advances in the underlying molecular mechanisms of regulated necrosis in the pathological process during cerebral ischemic preconditioning, perconditioning and postconditioning.
- In addition, we explore the crosstalk and interplay among the diverse types of regulated necrosis that contribute to ischemic stroke.

## Background

Ischemic stroke accounts for 87% of all strokes and is one of the most significant causes of neurological morbidity and mortality globally. This disease causes death and permanent disability in ~10 million people annually, and the risk of ischemic stroke continues to increase in low- and middle-income countries along with the accompanying rising economic and social burden for communities [1]. Ischemic stroke occurs when embolic or thrombotic events interrupt blood circulation to the brain. Although the primary treatment strategies for ischemic stroke are local recanalization and systemic thrombolysis to restore the blood supply as quickly as possible, these therapeutic options have a short therapeutic window within the initial 4.5 h, and only 20% of patients are eligible for these treatments [2]. To make matters worse, the reperfusion of blood could further contribute to neuronal injuries. Generally, the outcome of ischemic stroke is largely determined by the amount of ischemia- and reperfusion-related neuronal death in the infarct region.

The central nervous system (CNS), a major participant in cerebral ischemia–reperfusion, consists of various types of cells, including platelets, endothelial cells, astrocytes, neutrophils, microglia, macrophages and neurons. Due to their structures and functions, CNS cells have extremely high energy demands and are very sensitive to drops in oxygen and glucose due to blood flow interruption. Meanwhile, the cells are susceptible to injuries that are induced by reperfusion [3, 4]. Hence, the loss of CNS cells is the main pathogenesis of ischemic stroke. In recent years, studies have focused on potential neuroprotectants and a more thorough understanding of the mechanisms involved in the ischemic death process. However, a certain number of putative neuroprotectants have been investigated in preclinical models, but none have entered the clinical realm [5]. In the past, three cell death modes were used based on the morphological classification of cell death: apoptosis unassociated with digestion, autophagic cell death associated with autophagy, and heterophagy-related necrosis [6]. However, over the past two decades, numerous studies have focused on regulated necrosis, a type of necrosis that can be tightly regulated and is potentially druggable but nonapoptotic. Based on its morphological features, regulated necrosis is classified into different forms. As reported, almost all forms of regulated necrosis contribute to the development of ischemic stroke, e.g. necroptosis, pyroptosis, ferroptosis, pathanatos, mitochondrial permeability transition pore (mPTP)-mediated necrosis and oncosis [7–11].

As various CNS cells form an extensive communication network, neuronal cell death is a complex process, and regulated necrosis is involved in the death and dysfunction of cells. Except for morphological differences, there are specific molecular pathways for different forms of regulated necrosis. This review summarizes the current studies on the influence of regulated necrosis on the pathological process of ischemia/reperfusion-regulated injuries. The findings of this review may shed light on novel therapeutic targets for ischemic stroke.

## Review

### Influence of necroptosis on ischemic stroke

#### An overview of necroptosis in ischemic stroke

**Figure 1:**
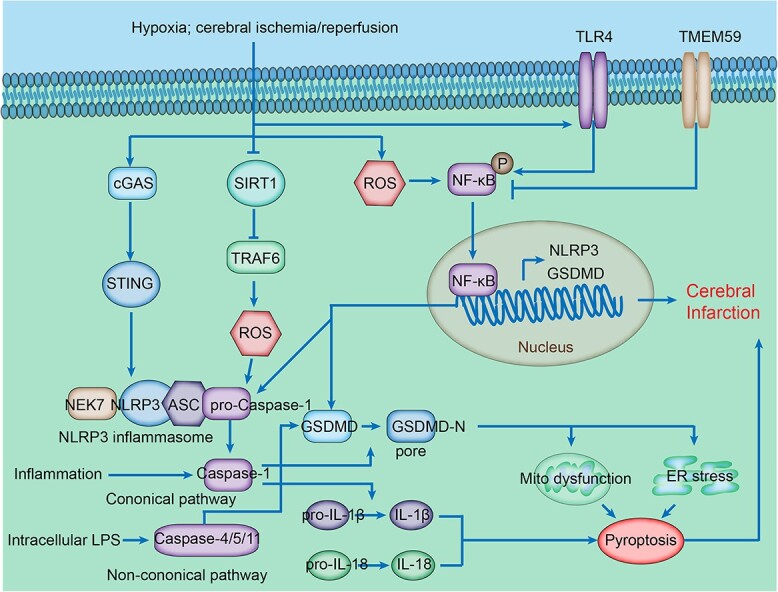
Necroptosis-related signaling pathway in ischemic stroke. Following cerebral ischemia, death ligands bind to death receptors and form complex I through recruiting TNFR1-associated death domain protein (TRADD), cellular inhibitor of apoptosis 1 and 2 (cIAP1/2), receptor-interacting protein kinase1 (RIPK1) and several E3 ubiquitin ligases. The polyubiquitinated RIPK1 in complex I can be deubiquitinated into RIPK1, on the one hand, forming pro-apoptotic complex IIa with TRADD, Fas-associating protein with a novel death domain (FADD) and caspase 8, and on the other hand, forming complex IIb through autophosphorylating itself to recruit and phosphorylate receptor-interacting protein kinase 3 (RIPK3), which subsequently recruits and phosphorylates mixed-lineage kinase domain-like pseudokinase (MLKL). Activated MLKL disrupts the cell membrane and cell lysis of cells and therefore executes necroptosis. Cerebral ischemia can stimulate necroptosis by activating ASIC1a/RIPK1 and TNFR1/RIPK3 signaling pathways. *TNFR* tumor necrosis factor receptor

Necroptosis is the best-characterized form of regulated necrosis. It is mediated by a cascade of kinases: receptor-interacting protein kinase 1 (RIPK1), RIPK3 and mixed-lineage kinase domain-like pseudokinase (MLKL). Upon receiving a necroptosis-inducing stimulus, RIPK1 phosphorylates and activates RIPK3, and the activated RIPK3 phosphorylates and activates MLKL in return, which finally forms a complex termed the necrosome [12]. Necrosome formation initiates necroptosis through plasma membrane disruption and cell lysis [12] (Figure 1). Thus, necroptosis has features of either apoptosis or necrosis. The characteristics of necroptosis are dependent on the kinase domain of RIPK3 that mediates MLKL phosphorylation. This process depends on the oligomerization of active RIPK3 molecules, which is mediated by the RIP homotypic interacting motifs (RHIMs) in RIPK1 and RIPK3 [13].

Death receptors (DRs), such as TNF receptor 1 (TNFR1), TNFR2, FAS (also known as CD95), TNF-related apoptosis-inducing ligand receptor 1 (TRAILR1) and TRAILR2, are well-studied initiators of necroptosis through downstream involvement of RIPK1, RIPK3 and MLKL [14–18]. The intracellular domains of TNFR1, FAS, TRAILR1 and TRAILR2 all include a death domain (DD). With the stimulation of their respective cognate ligands and the homotypic interactions between their DDs, they combine and form membrane-associated complex I with RIPK1, which contains a C-terminal death domain, an intermediate domain and an N-terminal kinase domain [16]. In complex I, RIPK1 is activated by multiple post-translational modifications, including ubiquitylation, deubiquitylation and phosphorylation. Furthermore, active RIPK1 plays an important role in apoptosis and necroptosis. The formation of a RIPK1–TNFR1-associated DD protein–FAS-associated DD protein–caspase 8 complex (complex IIa) leads to RIPK1-dependent apoptosis [19]. Then, activated RIPK1 interacts with and phosphorylates RIPK3, leading to the activation of RIPK3, which subsequently recruits and phosphorylates MLKL. Through forming a RIPK1–RIPK3–MLKL complex (complex IIb), MLKL phosphorylation disrupts the cell membrane, leads to cell lysis and executes necroptosis [20, 21]. Furthermore, due to the lack of cytoplasmic DD in TNFR2, TNFR2 triggers necroptosis by enhancing the cell’s sensitivity to endogenous TNF-induced TNFR1 pathways by depletion and/or degradation of TNF receptor associated factor 2 (TRAF2) [22]. Interestingly, in the absence of RIPK1, RIPK3 can be activated by TNF via TNFR1-associated death domain protein (TRADD) [23] and TRAF2. Activated RIPK3 then induces necroptosis through a caspase- and Bax/Bak-independent mechanism [24] (Figure 1).

Both initiators and mediators are widely expressed in various CNS cells, including neurons, microglia and platelets. Several studies have demonstrated that RIPK1, RIPK3 and MLKL participate in the pathogenesis of ischemic stroke after the activation of DRs. For example, mice with various RIPK1 kinase-dead knock-in mutations, RIPK3-knockout mice and MLKL-knockout mice showed normal development and adult life, indicating that necroptosis does not participate in the normal development of cells. Several mutant mice showed certain neuroprotective effects in diverse brain injuries.

RIPK1 inhibitors exert protective effects in ischemic stroke, further revealing the contribution of necroptosis to ischemic stroke [8, 15]. Compared with wild-type mice, RIPK1^K45A/K45A^ and RIPK1^D138N/D138N^ mice demonstrated less neuronal cell death and neurological dysfunction 24 h after intracerebral hemorrhage [25]. Treatment with RIPK1-specific inhibitors such as necrostatin-1 (Nec-1), GSK963 and 5-(3′,5 dimethoxybenzal)-2-thio-imidazole-4-ketone (DTIO) also reduced infarct volume and neurological deficit scores and significantly decreased ischemia-induced neuroinflammation and generation of reactive oxygen species (ROS) [15,25–27]. RIPK3 deficiency attenuated the cognitive dysfunction, inflammation and oxidative stress induced by traumatic brain injury [28]. Based on these results, necroptosis can drastically alter neuronal homeostasis, and targeting the RIPK1/RIPK3/MLKL pathway could be a viable treatment strategy against brain injuries.

Since necroptosis was defined in 2005, numerous studies have been dedicated to investigating the activation mechanism and regulation of necrosomes for their extensive expression in almost all cell types after stress stimuli and their close correlation to a series of cell loss-related diseases [15]. By intervening in the RIPK1/RIPK3/MLKL pathway, previous studies have demonstrated that necroptosis contributes to ischemic stroke. The protein levels of RIPK1 MLKL and pMLKL are significantly increased in stroke tissues of both rats [8] and human beings [29]. In the middle cerebral artery occlusion (MCAO) model *in vivo* and the oxygen–glucose deprivation/re-oxygenation (OGD/R) cellular model *in vitro*, blocking the RIPK1/RIPK3/MLKL pathway could effectively alleviate ischemic stroke injuries [15, 30].

Next, several studies have revealed that multiple post-translational modification molecules, such as cylindromatosis (CYLD), chromatin immunoprecipitation (CHIP), Triad3A and ribosomal S6 kinase 3 (RSK3), have been implicated in stroke by mediating the activation of RIPK1 and RKPK3 [31–34]. In this paper, we focus on findings about necroptosis in different cell types in ischemic stroke, including neurons, astrocytes, platelets, microglial cells and endothelial cells.

#### Necroptosis in neurons and microglia following ischemic stroke

Due to high energy demands and the inability to perform mitosis, neurons are sensitive to interruptions in blood flow and are the first brain cells to die in ischemic stroke [35]. Neuronal death and loss of interaction between neurons and microglia further induce neuroinflammation [36] and intensify ischemic stroke damage [37]. In previous studies, necroptosis was found to contribute to ischemic stroke by intervening in the RIPK1/RIPK3/MLKL signaling pathway in neurons and microglia. In the MCAO rat model, cerebral ischemia stimulated RIPK1 phosphorylation at the Ser166 residue, RIPK3 phosphorylation at the Ser232 residue and MLKL phosphorylation at the Ser345 residue and significantly increased RIPK1, RIPK3 and MLKL levels, while Nec-1 attenuated these changes by inhibiting phosphorylated RIPK1 kinase [8]. Similarly, a murine endothelin-1 model of ischemia–reperfusion injury exhibited an increase in necroptosis. Furthermore, with Nec-1 treatment, RIPK1 inhibition reduces the activation of both caspase-3 and caspase-7, revealing a new form of interaction between apoptosis and necroptosis [38]. RIPK1 is activated upstream of RIPK3 after ischemic insult, and RIPK3 deficiency reduces long-term ischemic infarcts [39]. Since the expression of TNFR1 and TNFR2 is quite low in normal neurons [40], certain studies have focused on the various activators of the RIPK1/RIPK3/MLKL pathway in addition to TNFα signaling.

Upon TNFα stimulation, RIPK1 is recruited into complex I together with several adaptor proteins, such as TRADD, TRAF2 and cellular inhibitor of apoptosis 1 and 2 (cIAP1/2). TRAF2 is one of the family members of TRAF. It typically shares a common TRAF domain and can bind to TRADD, the adaptor protein. Although TRAF2 has a RING E3 ubiquitin ligase domain, multiple reports have confirmed that TRAF2 participates in the K48- and K63-linked polyubiquitination of RIPKI by binding to cIAP1 and cIAP2 [41, 42] and acts as a necroptosis suppressor through different mechanisms [43]. Ischemic stroke has been shown to induce the expression of TRAF2 in neurons and microglia. Through the formation of a complex with MLKL, TRAF2 protects microglia and neurons against necroptosis, and the knockdown of TRAF2 amplifies infarct volume and increases cell death via the necroptosis mechanism [44]. Triad3A is also involved in ischemic insult. The E3-ubiquitin ligase Triad3A induces the early degradation of necrosomes by promoting the K48-ubiquitin-dependent proteasomal degradation of RIPK1 [45]. Therefore, in the mouse MCAO model, Triad3A was markedly induced, especially in neurons and microglia. Additionally, Triad3A knockdown accelerated cell death, increased microglial activity, enhanced the infarction area and promoted the expression of pro-inflammatory markers, including TNF-α, interleukin (IL)-1β and inducible nitric oxide synthase (iNOS) [33]. Remarkably, transforming growth factor β-activated kinase 1 (TAK1, also called MAP3K7), which is a member of the mitogen-activated protein kinase (MAPK) family, plays an important role in the phosphorylation of RIPK1 at multiple sites [46]. Several studies have proven that TAK1 is rapidly activated in ischemic stroke, and inhibition of TAK1 both via conditional knockout and via treatment with 5Z-7-oxozeaenol can reduce infarct volume and cell death and improve neurological outcomes [47, 48]. Naito *et al*.’s study showed that the conditional knockout of TAK1 in microglia/infiltrated macrophages and neuronal lineages aggravated neuronal death and neuroinflammation after ischemic insult through the transition from early necroptosis to apoptosis [39].

These contradictory results may be related to the inhibition term length of TAK1. Neubert *et al*. demonstrated that the short-term and brief inhibition of TAK1 exerted a neuroprotective effect by interfering with the activation of p38/MAPK and JNK in experimental stroke models. Conversely, prolonged inhibition of TAK1 induced the upregulation of apoptosis signal-regulating kinase-1 (ASK-1), which can offset TAK1 inhibition [49].

In addition to the TNF-associated signaling molecules mentioned above, acidotoxicity, common in ischemic stroke, can induce neuronal necroptosis via acid-sensing ion channel 1a (ASIC1a) [50]. In an experimental stroke model, independent of its ion-conducting function, ASIC1a was significantly induced and participated in the phosphorylation of RIPK1 by recruiting RIPK1 to the ASIC1a C-terminus (CT) and mediated RIPK1 activation. In addition, the N-terminus (NT) of ASIC1a auto-inhibits the CT to prevent RIPK1 recruitment/activation under resting conditions. As a consequence, both expression of mutant ASC1a and treatment with N-ethylmaleimide-sensitive fusion ATPase (NSF), which associates with ASIC1a-NT under acidosis, could cause neuronal death, whereas in the presence of a membrane-penetrating synthetic peptide representing the distal 20 ASIC1a NT residues, neuronal damage was reduced in experimental stroke [51]. These results reveal novel strategies for preventing neuronal death and neuroinflammation by targeting the necroptosis pathway.

#### Necroptosis in astrocytes following ischemic stroke

The prominent pathological features of ischemic stroke include reactive astrogliosis and glial scar formation [52]. A glial scar mainly consists of astrocytes, the most abundant cell type in the CNS. The scar is characterized by cellular hypertrophy, proliferation and increased expression of the intermediate filament protein [53].

Increasing evidence attests that depending on brain injury time, reactive astrocytes perform both beneficial and/or detrimental functions. In acute ischemic stroke, reactive astrocytes not only secrete anti-inflammatory factors and take up excess glutamate but also release various toxic molecules, including ROS, cytokines and Vascular endothelial growth factor (VEGF) [54, 55]. In the chronic phase after ischemic stroke, reactive astrocytes express a broad range of inhibitory molecules against axonal regeneration, such as chondroitin sulfate proteoglycans (CSPGs), which inhibit axonal regeneration [52]. In an ischemic stroke study performed by Zhu *et al*., activation of the RIPK1/RIPK3/MLKL pathway contributed to astrogliosis and glial scar formation by enhancing VEGF-D/vascular endothelial growth factor receptor (VEGFR)-3 signaling, and Nec-1 attenuated brain damage by inhibiting the formation of astrogliosis and glial scar via suppression of the RIPK1 and VEGF-D/VEGFR-3 signaling pathways [56]. Similar results were obtained in experimental stroke models; both Nec-1 and SB216763 (a glycogen synthase kinase-3 β inhibitor) attenuated ischemia-induced reactive astrogliosis by reducing the interaction between RIPK1 and glycogen synthase kinase 3β (GSK3β) and inhibiting inflammatory cytokines [57]. The above-mentioned studies support the contribution of necroptosis to ischemic stroke-induced reactive astrogliosis and glial scar formation.

#### Necroptosis in platelets following ischemic stroke

Platelets, which are produced from megakaryocytes, are essential for thrombus formation. As a result, platelets are the primary target of classic anti-platelet agents in ischemic stroke [58]. RIPK3 is known to be a novel modulator of platelet activation through its interaction with Galpha 13 (Gα13) in platelets; thus, RIPK3 deficiency leads to the inhibition of platelet aggregation and thrombus formation [59].

Emerging evidence highlights that platelets contain membrane receptors that detect pathogen- and danger-associated molecular patterns (PAMPs and DAMPs, respectively). Furthermore, platelets mediate heterotypic leukocyte interactions and recruitment, which promote brain injury following ischemic stroke [60]. Recently, necroptosis signaling proteins were suggested to play a role in venous thrombosis based on the effects of RIPK1 and MLKL in modulating neutrophil–platelet aggregation [61]. Reduced neutrophil death and neutrophil–platelet aggregation were evident with Nec-1 and genetic deletion of MLKL; these effects were linked to reduced neutrophil necroptosis induced by activated platelets but not platelet activation itself. In addition, TNFR1 expression in platelets is rather low [62], and both the role and the execution pathway of platelet necroptosis in ischemic stroke still demand more research.

#### Necroptosis in endothelial cells following ischemic stroke

Disruption of the blood–brain barrier (BBB) after ischemic stroke contributes to further development and enlargement of brain injury [63]. Endothelial cells are an important component of both the BBB and the neurovascular unit, and the death of endothelial cells contributes to vascular injury and BBB disruption in ischemic stroke [64]. The role of necroptosis signaling proteins in BBB disruption was demonstrated based on the effects of RIPK1 and MLKL, which were induced by microglia-secreted TNF-α in regulating endothelial cell necroptosis [65]. Decreases in BBB leakage and brain injuries were evident with Nec-1 and anti-TNFα (infliximab) and linked to reduced endothelial necroptosis and microglia-secreted TNF-α. Since endothelial necroptosis is rapidly activated after a transient ischemic insult, more studies are needed to explore the role of endothelial necroptosis in ischemic stroke.

### Influence of pyroptosis on ischemic stroke

#### An overview of pyroptosis in ischemic stroke

**Figure 2:**
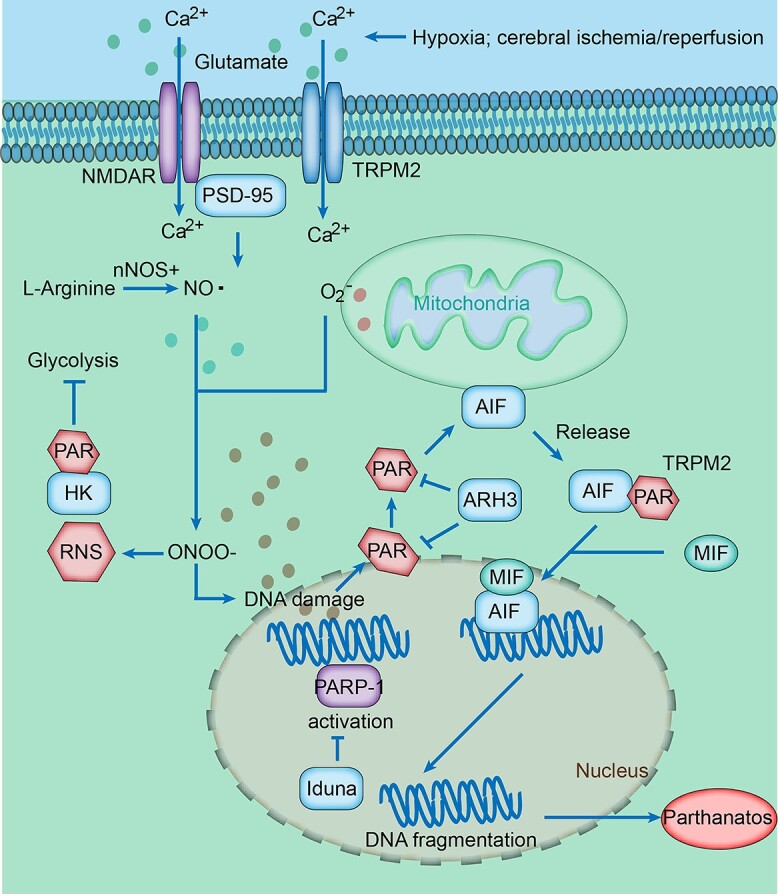
Pyroptosis signaling pathway in ischemic stroke. After ischemic stroke, NLRP3 inflammasome is activated through several mechanisms, including the activation of the cGAS/STING, ROS/NF-κB and TLR4/NF-κB pathways, and the inhibition of the SIRT1/TRAF6 and TMEM59/NF-κB pathways, and contributes to caspase-1 activation. Activated caspase-1 cleaves the hinge region between the active N- and inhibitory C-terminal domains of gasdermin-D (GSDMD) and converts pro-interleukin (IL)-1β and pro-IL-18 into their active forms. GSDMD-N translocates to the plasma membrane and organelle membranes and forms membrane pores, which could induce pyroptosis. In the non-canonical pathway, intracellular lipopolysaccharide causes activation of caspase-4/5/11, leading to GSDMD cleavage. *ROS* reactive oxygen species, *NF-κB* nuclear factor kappa-B

Pyroptosis, gasdermin-mediated programmed necrosis, is characterized by rapid plasma membrane rupture and extracellular release of inflammatory cytokines. Traditionally defined through its binding with nitric oxide dioxygenase (NOD)-like receptor family pyrin domain-containing 3 (NLRP3) and apoptosis-associated speck-like protein containing a CARD (ASC), caspase-1 cleaves and activates gasdermin-D (GSDMD) and evokes GSDMD-executed pyroptosis cell death [66]. Its lytic and inflammatory type of programmed cell death is characterized by the maturity and release of pro-inflammatory mediators, including IL-1β and IL-18. This progress relies on the activation of pro-inflammatory caspases such as the canonical pathway of caspase-1 and the noncanonical pathway involving caspase-4,5 (in humans), caspase-11 (in mice) and gasdermin protein cleavage (Figure 2). An intense inflammatory response occurs immediately after ischemic stroke. The DAMPs released by dead cells are recognized by pattern recognition receptors (PRRs) and lead to the formation of inflammasomes [67]. Four known inflammasome-forming proteins, namely, NOD-like receptor family pyrin domain-containing 1 (NLRP1), NLRP3, absent in melanoma 2 (AIM2) and NOD-like receptor family caspase recruitment domain protein (CARD) domain-containing 4 (NLRC4), have been confirmed as inflammasome sensors in ischemic stroke with highlighted upregulation [68]. In the canonical pathway of caspase-1-mediated pyroptosis, caspase-1 is recruited to inflammasome sensor proteins via the interaction of the CARD domain with ASC. Caspase-1 is also self-activated by proteolytic cleavage to its catalytically active species, the p10/p20 tetramer [69, 70]. On the one hand, activated caspase-1 recognizes and converts IL-1β and IL-18. In contrast, activated caspase-1 cleaves the hinge region between the N- and C-terminal domains of GSDMD. Then, it oligomerizes GSDMD-N, which contains a pore-forming domain to mediate the formation of membrane pores with a diameter of ~10–14 nm in the plasma membrane [71]. Apart from caspase-1, caspase-4 and caspase-5/11, activated by lipopolysaccharide, cleave GSDMD and initiate pyroptosis in the non-canonical pathway, but without producing IL-1β and IL-18 [72, 73]. Moreover, similar to GSDMD, GSDME (originally identified as deafness autosomal dominant 5, DFNA5) contains a pore-forming domain in the N-terminal which is specifically cleaved by caspase-3 to form a pyroptosis pore in the plasma membrane and switches the activation of caspase-3 from driving an apoptotic program to causing pyroptosis [74]. In addition, GSDME is a transcriptional target of p53, indicating its novel cellular function in the stress response [75].

In 2001, pyroptosis was established as pro-inflammatory programmed cell death in the immune, central nervous and cardiovascular systems [76]. As a result, researchers of pyroptosis began to focus on CNS diseases. Pyroptosis contributes to neuroinflammation and neurodegeneration and aggravates brain tissue damage in ischemic stroke by directly promoting the pyroptotic cell death of neurons, endothelial cells and glial cells [77]. Emerging evidence has shown that pyroptosis-related molecules, including NLRP1, NLRP3, NLRC4, AIM2 and caspase-1, are significantly elevated after cerebral ischemic injury [78–80]. In the MCAO rat model, GSDMD, a key executor of pyroptosis, immediately increased after ischemic insult and then peaked at 24–48 h post-ischemia [81]. Accumulated evidence has shown that the inhibition of pyroptosis via pharmacologic intervention or gene knock-down of pyroptosis-related molecules such as multiple inflammasomes, caspase-1/3/4/5/11 and gasdermin family proteins has neuroprotective effects against cerebral ischemic stroke injuries [82–85]. Additionally, a number of studies have revealed that multiple pathways, such as nucleic acid-sensing cyclic GMP-AMP synthase (cGAS), nuclear factor-kappaB (NF-κB) and triggering receptor expressed on myeloid cells-1 (TREM-1), are involved in stroke via the activation of inflammasomes (Figure 2).

#### Pyroptosis in microglia following ischemic stroke

As the primary immune defense cells of the brain, microglia can differentiate into either a classical pro-inflammatory state (M1) or an alternative anti-inflammatory state (M2) in response to ischemic stroke insult [86]. As M1 phenotype microglia release pro-inflammatory cytokines, mounting evidence has verified that cerebral ischemic injury-induced neuroinflammation and pyroptosis can be attenuated by modulating microglial phenotypes.

TREM-1, a transmembrane immune receptor, is expressed in most innate immune cells. It is a potent amplifier of inflammation by playing an important role in microglial pyroptosis [87]. Xu *et al*. found that the activation of TREM-1 was accompanied by microglial M1 polarization following cerebral ischemic injury through interactions with spleen tyrosine kinase (SYK) and elevated levels of GSDMD and GSDMD-N [88]. Remarkably, pharmacologic inhibition of TREM-1 with the synthetic peptide LP17 abrogated microglial M1 polarization and the activation of pyroptosis-related molecules, improving stroke outcomes [88, 89]. Similarly, TMEM59 (also known as dendritic cell-derived factor 1), a type I transmembrane protein, plays a crucial role in neuroinflammation [90]. Liu *et al*. found that TMEM59 could interact with a triggering receptor expressed on myeloid cells 2 (TREM2). In turn, this interaction mediateed the degradation of TMEM59, and the knockdown of TMEM59 promoted microglial activities [91]. In Zhang *et al*.’s study, knockdown of TMEM59 dramatically potentiated microglial activation, pyroptosis and neurological injury after ischemic insult via activation of the NF-κB signaling pathway [92].

In addition, cGAS is a key cytosolic DNA sensor that is significantly upregulated along with microglial activation and pyroptosis by interacting with stimulators of interferon genes (STING) after experimental ischemic stroke, while A151 could effectively attenuate these changes by inhibiting the cGAS–STING pathway [93]. In addition to pharmacologic intervention or gene knockdown strategies, exosomes exhibit protective effects against cerebral ischemia-induced pyroptosis [94, 95]. Overall, these findings reflect the therapeutic potential of the microglial pyroptosis pathway in treating ischemic stroke.

#### Pyroptosis in neurons following ischemic stroke

As mentioned above, the loss of neurons is the core problem in stroke; meanwhile, the death of neurons can lead to the death of other neurons connected to the dying neurons [96]. The evidence for the role of pyroptosis in stroke-induced neuronal death has been well defined in several studies. In MCAO mice, the nicotinamide adenine dinucleotide-dependent deacetylase Sirtuin 1 (Sirt1) reduced ischemic stroke-induced neuronal pyroptosis by inhibiting ROS and tumor necrosis factor receptor associated factor 6 (TRAF6) in hippocampal neurons [9], while valproic acid countered neuronal pyroptosis via apoptosis repressor with caspase recruitment domain (ARC)/caspase-1/GSDMD axis in cerebral ischemic injury [97]. In another study, low-density lipoprotein receptor (LDLR), which is important for cholesterol uptake, suppressed neuronal pyroptosis by inhibiting the NLRP3/ASC/caspase-1 pathway following ischemic stroke in mice. Moreover, administration of CY-09 (a inhibitor of NLRP3) substantially deferred neuronal pyroptosis in Ldlr^−/−^ stroke mice [98]. In addition, the long non-coding RNAs maternally expressed gene 3 (MEG3) was upregulated in experimental stroke *in vivo* and *in vitro* by sponging miR-485 and abrogating the suppression of AIM2. Thus, knockdown of MEG3 could inhibit neuronal pyroptosis by repressing AIM2/ASC/caspase-1 signaling [99]. Furthermore, Zhao *et al*. found that knockdown of platelet-activating factor receptor (PAFR) via XQ-1H (a specific PAFR inhibitor) or small interfering RNA (siRNA) mitigated neuronal pyroptosis by suppressing the NLRP3/ASC/caspase-1 axis following cerebral ischemic stroke [100]. Therefore, targeting these regulatory pathways in neuronal pyroptosis may be a novel therapeutic strategy for treating ischemic stroke.

Pyroptosis in endothelial cells can further contribute to ischemia-induced BBB dysfunction. Liang *et al*. demonstrated that administration of vx-765 (a specific caspase-1 inhibitor) ameliorated the activation of ischemia-induced pyroptosis and restored the permeability of the BBB by inhibiting the receptor for advanced glycation end products/MAPK pathway [101]. Additionally, medioresinol attenuated the pyroptosis of brain microvascular endothelial cells in ischemic stroke as a novel PGC-1α activator by promoting the PPARα/GOT1 axis [102]. Moreover, pyroptosis of astrocytes and microglia is able to trigger the accumulation of amyloid-beta peptide (Aβ) and BBB dysfunction in ischemic stroke by inhibiting the polarization of aquaporin-4 (AQP-4) [103].

#### Influence of ferroptosis on ischemic stroke

**Figure 3:**
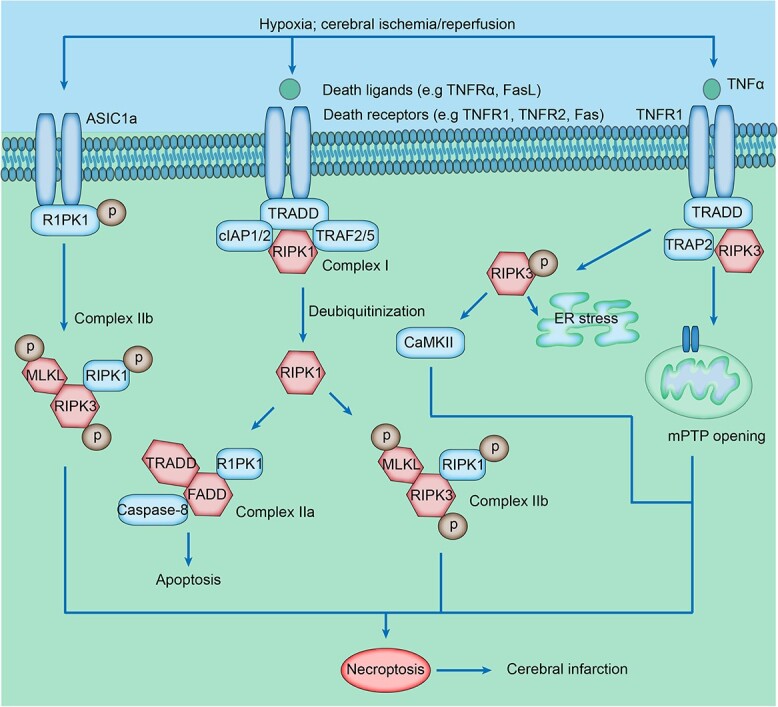
Ferroptosis-related signaling pathway during the progression of ischemic stroke. During the onset of cerebral ischemia ischemic stroke, impaired system x_c_− causes suppression of cystine–glutamate exchange and deficiency of glutathione (GSH) and glutathione peroxidase 4 (GPX4), accumulating toxic lipid reactive oxygen species (ROS). Increased intracellular Fe^3+^ is transported to cytoplasm and reduced to Fe^2+^ with the synergy of the six-transmem brane epithelial antigen of prostate 3 (STEAP3). Abundant Fe^2+^ accelerates the generation of lipid ROS through Fenton reaction. In addition, polyunsaturated fatty acids consumption, with the cooperation of acyl-CoA synthetase long-chain family member 4 (ACSL4) and Fenton reaction, results in ferroptosis. Nuclear receptor coactivator 4 (NCOA4) and USP14/NCOA4 pathways also promote ferroptosis by inhibiting ferritinophagy

Ferroptosis is iron-regulated necrosis characterized by iron overload or lipid peroxidation accumulation. It is launched by four crucial signaling methods: (1) iron overload, (2) lipid peroxidation, (3) impaired system x_c_^−^ and (4) depletion of glutathione (GSH) and inactivation of glutathione peroxidase 4 (Gpx4) [104, 105] (Figure 3). As the basic structure of cell membranes, polyunsaturated lipids are at high risk of lipid peroxidation, especially in the state of abundant Fe(II) and Fe(II)-dependent enzymes, and the membranes turn into a provider of damaging reactive lipid peroxides [106] (Figure 3). Therefore, ferroptosis is defined by the accumulation of iron-dependent lipid-ROS and the consumption of cell membrane polyunsaturated fatty acids (PUFAs) [107, 108].

GSH is necessary to prevent lipid-ROS accumulation by participating in reactions catalyzed by GPX4, GSH-dependent glutathione transferases (GSTs) and GPXs [109, 110]. Moreover, GSH synthesis requires consecutive import of cystine–cystine disulfide (Cys_2_) by system x_c_^−,^ which serves as a Cys_2_/glutamate antiporter [111]. Hence, as mentioned before, system x_c_^−^ inhibitors (glutamate, erastin and sorafenib), GPX4 inhibitors (RSL3 and FIN56) and GSH inhibitors (buthioninesulfoximine and acetaminophen) can all induce ferroptosis [107, 112, 113]. Interestingly, GSH depletion and/or GPX4 inhibition fail to induce ferroptosis without incorporating PUFAs into membrane phospholipids [114].

In the CNS, iron catalyzes the generation of hydroxyl radicals through the Fenton reaction and inducing oxidative stress [115]. As early as the 1990s, it was already known that iron overload accelerates neuronal damage both in clinical and experimental models of ischemic stroke, while iron depletion attenuates ischemia impairment [116, 117]. Tuo *et al*. first confirmed that ferroptosis inhibitors (liproxstatin-1 and ferrostatin-1) attenuated ischemia–reperfusion injury in the MCAO mouse model. Moreover, they found that tau depletion protected against MCAO-induced ferroptosis in young mice and that iron-targeting interventions contributed to restoring the protective effect of tau depletion in an MCAO model of aged mice, indicating that ferroptosis is involved in ischemia-induced neuronal death. The tau–iron interaction can be a multi-effect modulator of ferroptosis and ischemic stroke [118]. In a study performed by Cui *et al*. [119], acyl-CoA synthetase long-chain family member 4 (ACSL4) overexpression exacerbated neuronal ferroptosis and the release of pro-inflammatory cytokines in microglia by catalyzing the esterification of arachidonoyl and adrenoyl into phosphatidylethanolamine. Thus, interventions involving the ACSL4 pathway in defending against ferroptosis might provide a novel therapeutic strategy for ischemic stroke [119, 120].

Another study demonstrated that the downregulation of light-chain subunit solute carrier family 7 member 11 (SLC7A11), a multi-pass transmembrane protein that serves as the glutamate–cystine antiporter in system x_c_^−^, resulted in neuronal ferroptosis following OGD/R stimuli in primary mouse cortical neurons. Treatment with kaempferol could reverse OGD/R-induced ferroptosis by accelerating the expression of SLC7A11 [121].

In addition, p53 promotes ferroptosis by inhibiting the expression of SLC7A11. Lu *et al*.’s study indicated that miR-214 downregulation was correlated with neuronal ferroptosis in acute ischemic stroke patients by abrogating the suppression of p53, while long non-coding RNA PVT1 promoted ischemic injury by binding to miR-214 [122]. Li *et al*. demonstrated that the upregulation of nuclear receptor coactivator 4 (NCOA4) in the cytoplasm, a selective cargo receptor mediating ferritinophagy, promoted neuronal ferroptosis in an MCAO mouse model, and the knockdown of NCOA4 protected neurons from ferroptosis by blocking ferritinophagy. Moreover, USP14 was identified as the deubiquitinating enzyme (DUB) to cleave ubiquitin from NCOA4, and USP14 inhibition had neuroprotective effects by suppressing NCOA4-induced ferroptosis [123].

As mentioned above, liproxstatin-1 and ferrostatin-1 are the typically acknowledged inhibitors of ferroptosis and have been reported to alleviate cerebral ischemia/reperfusion injury in rats [118]. Multiple iron chelators, such as deferoxamine, deferasirox and dexrazoxane, have shown anti-ferroptotic effects in clinics and experimental stroke models [124–126]. Additionally, certain herbal compounds, such as carvacrol and kaempferol, protect neurons from cerebral ischemia-induced ferroptosis in experimental stroke models by increasing the expression of GPX4 [121, 127]. Electroacupuncture, a common treatment in the clinic to improve the neurological function and quality of life of ischemic stroke patients, can suppress neuronal ferroptosis by accelerating the expression of GPX4 and ferritin heavy chain 1 (FTH1) in MCAO rats [128]. Thus, ferroptosis inhibition could potentially improve ischemic stroke outcomes in the clinic.

#### Influence of parthanatos on ischemic stroke

**Figure 4:**
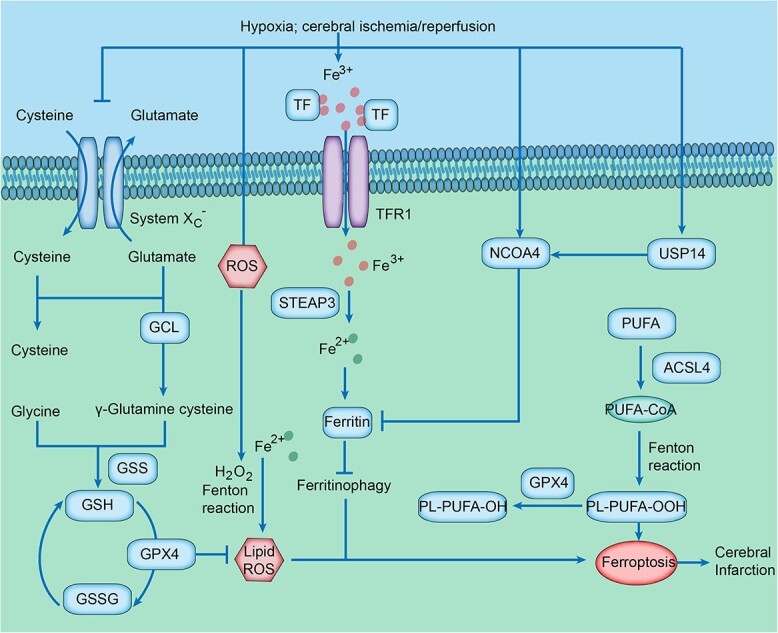
Parthanatos signaling pathway in ischemic stroke. During the progression of ischemic stroke, increased intracellular Ca^2+^ binds to calmodulin and causes abundant ONOO^−^, stimulating DNA damage. Poly-(ADP-ribose) polymerase 1 (PARP1) is over-activated by DNA damage and generates poly-(ADP-ribose) (PAR). The translocation of PAR polymer from the nucleus to mitochondria causes the release of apoptosis inducing factor (AIF) from mitochondria and contributes to the formation of AIF/MIF complex. Subsequently, AIF/MIF complex translocates to the nucleus and generates DNA fragmentation, leading to parthanatos. In addition, PAR polymer can inhibit glycolysis via binding HK. Iduna and ADP-ribose-acceptor hydrolase 3 (ARH3) also inhibit parthanatos by mediating the ubiquitination of PARP1 and decreasing PAR accumulation in both cytoplasm and nucleus

Parthanatos is a poly-(ADP-ribose) polymerase 1 (PARP1)-dependent necrotic cell death and is triggered by excessive DNA damage. PARP1 overactivation leads to the accumulation of poly-(ADP-ribose) (PAR) polymers in the nucleus and results in the depletion of NAD^+^ and ATP. The depletion of NAD^+^ and free PAR polymer can cause the release of apoptosis inducing factor (AIF) from mitochondria and nuclear translocation of the AIF/MIF complex and finally generate chromatin condensation and large scale DNA fragmentation (~50 kb) leading to cell death [129, 130] (Figure 4). Intervention in the form of PARP inhibitors and knockdown of PARP1 can effectively suppress AIF release and cell death [131]. Multiple mechanisms, including oxidative stress, nitrosative stress, inflammation, hypoxia, ischemia and DNA-alkylating agents, contribute to the pathological activation of PARP1. Recent studies suggest that parthanatos plays a crucial role in neuronal cell death in ischemic stroke.

In 1994, Zhang *et al*. revealed that activation of PARP by treatment with N-methyl-D-aspartic acid (NMDA) or high levels of NO contributed to cell death in rat cerebral cortical neurons, and suppression of PARP effectively defended against NMDA neurotoxicity [132]. Eliasson *et al*. reported that gene disruption of PARP could abrogate glutamate–NO-mediated neurotoxicity in both cortical neurons and an MCAO rat model, suggesting that inhibition of PARP may provide a novel therapeutic strategy in ischemic stroke [133]. Consistent with these findings, various studies have found that the accumulation of PAR and the expression of PARP1 are significantly upregulated in ischemic brain tissue [134, 135], and PARP1 inhibitors such as 3-amino benzamide, 3,4-dihydro-5-[4–1(1-piperidinyl) butoxy]-1(2H)-isoquinolinone, and thieno[2,3-c]isoquinolin-5-one provide significant neuroprotection in experimental models of ischemic stroke *in vivo* and *in vitro* [136–138]. JPI-289 (also called amelparib), a PARP1 inhibitor, has also shown effective neuroprotection in experimental models of ischemic stroke *in vivo* and *in vitro* [139]. Moreover, the safety, tolerability and pharmacokinetics of JP1–289 were evaluated in healthy Korean male volunteers through single ascending dose and multiple ascending dose studies [140].

By hydrolyzing PAR polymers to free ADP-ribose units by its endo- and exo-glycosidase activities, PAR glycohydrolase (PARG) acts as a crucial enzyme in the reactivation of automodified PARP1 and plays an important role in hyper-poly(ADP-ribosyl)ation, contributing to neuronal death [141]. Therefore, PARG inhibition could lead to the accumulation of PARP1 auto-poly(ADP-ribosyl)ation and suppression of PARP1 hyperactivation. Multiple PARG inhibitors, such as gallotannin, nobotanin B and *N*-bis-(3-phenyl-propyl)9-oso-fluorene-2,7-diamide, have demonstrated remarkable neuroprotection against PARP1-dependent cell death in ischemic brain damage [141, 142]. However, although ADP-ribose-acceptor hydrolase 3 (ARH3) is crucial in PAR degradation, it contributes to cleaving ADP-ribosylated serine by hydrolyzing PAR and *O*-acetyl-ADP-ribose and decreasing PAR accumulation in the cytoplasm and nucleus [143] (Figure 4). Depletion of ARH3 leads to PAR accumulation in the cytoplasm and AIF translocation to the nucleus and increases sensitivity to parthanatos in cerebral ischemia/reperfusion injury [144, 145]. Moreover, intervention with PARP1 inhibitors assuaged ischemic brain damage through lowering parthanatos in ARH3-deficient mice [145].

Iduna is a PAR-dependent ubiquitin E3 ligase, and it mediates the ubiquitination and proteasomal degradation of PARP1 in turn [146] (Figure 4). Overexpression of Iduna alleviates cerebral ischemic injury by reducing PAR polymer and AIF accumulation [147]. Targeting the PAR/AIF pathway, Danhong injection, astragaloside IV and baicalein inhibited parthanatos, eliminated neurological dysfunction and ameliorated cerebral ischemia injury *in vivo* and *in vitro* [148–150]. Further study also revealed that co-culturing with mesenchymal stem cells protected cortical neurons against OGD-induced parthanatos by inhibiting AIF nuclear translocation [151]. Furthermore, the overproduction of ROS and increasing calcium (Ca^2+^) are known as essential messengers in cell death, and these can result in PARP1 activation and parthanatos [152, 153]. 14,15-Epoxyeicosatrienoic acid attenuated ischemia-induced parthanatos through reducing the generation of ROS in a MCAO rat model [154]. The endoplasmic reticulum (ER) and mitochondria are the cores of ROS production and Ca^2+^ release and are involved in parthanatos. In Zhong *et al*.’s study, propofol diminished infarct volume and enhanced neurological score by restraining ischemia-induced parthanatos via suppression of ROS and Ca^2+^ release in a MCAO mouse model [155]. Therefore, targeting the mediators of parthanatos may be a potential strategy for treating ischemic stroke.

#### Influence of mPTP-mediated necrosis on ischemic stroke

The mitochondrial permeability transition pore (mPTP) is the pore formed in the inner mitochondrial membrane (IMM). Induced by oxidative stress and cytosolic/mitochondrial Ca^2+^ accumulation, the mPTP is responsible for both apoptotic and necrotic cell death. The pores have a diameter of ~1.4–2.3 nm and render the IMM freely permeable to molecules up to 1.5 kDa in size, resulting in loss of mitochondrial membrane potential, expansion of the matrix and rupture of the mitochondrial outer membrane [156].

Cyclophilin D (CypD) is a peptidyl-prolyl *cis*–*trans* isomerase in the mitochondrial matrix and mediates mPTP opening depending on its sensitivity to Ca^2+^ [157]. CypD is an essential mediator in mPTP-mediated necrosis. In Schinzel *et al*.’s study, CypD knockout produced effective resistance against cerebral ischemia/hypoxia-induced mitochondrial dysfunction, oxidative stress and Ca^2+^ overload *in vivo* and *in vitro* [157]. Denorme *et al*. found that mice with CypD-deficient platelets demonstrated fewer neutrophils and platelet–neutrophil aggregates, significant enhancement of cerebral blood flow, improvement of neurological and motor functions and decreased infarct volume in ischemic stroke, indicating that CypD is a crucial mediator in mPTP-mediated necrosis in ischemic stroke [158]. The immunosuppressant drug cyclosporin A (CsA) serves as an inhibitor of cyclophilins. It exhibits neuroprotective effects on cerebral ischemia/reperfusion-treated brains and hypoxia/reoxygenation-treated neurons by inhibiting mPTP opening and interacting with CypD [159–161].

Further studies have found that multiple post-translational modifications, including phosphorylation, deacetylation, ubiquitination and *S*-nitrosylation of CypD, could regulate mPTP opening and cell death. GSK3β translocates from the cytosol to mitochondria to induce mPTP opening by interacting with CypD, and GSK3β inhibition exerts neuroprotective effects that attenuate mitochondrial dysfunction and ameliorate infarct volume in the MCAO mouse model [162, 163]. Sirt3 has been proven to deacetylate CypD in various types of cells, and Sirt3 overexpression protects neurons against metabolic and excitotoxicity stress [164]. Furthermore, several studies have reported that Sirt3 overexpression suppresses cerebral ischemia-induced mPTP opening [165–167], but the connection between its neuroprotective effects on ischemic stroke and the deacetylation of CypD is still unclear. In addition, p53 has been reported to interact with CypD and trigger mPTP-mediated necrosis of neurons in ischemic stroke [168], and p53 deficiency could remarkably suppress mPTP opening in OGD/R-treated neurons [160]. Until now, the structure and components of the mPTP have not been entirely clear; hence, more studies are needed to elucidate the signaling pathways of the mPTP. To summarize, mPTP-mediated necrosis could be a potential therapeutic target in ischemic stroke.

#### Influence of oncosis on ischemic stroke

Oncosis is characterized by cellular swelling, organelle swelling and blebbing, and increased membrane permeability. It is usually triggered by ischemia and toxic agents through interruption of plasma membrane ionic pumps [169]. Various CNS cell types, such as neurons, glia and vascular endothelial cells, suffer oncosis and manifest as cytotoxic oedema and vasogenic oedema in ischemic stroke [170, 171]. The depletion of ATP and ionic dyshomeostasis (Ca^2+^, Na^+^ and Cl^−^ influx and K^+^ efflux) induced by sudden termination of blood flow have been found to contribute to the oncotic process in ischemic stroke.

Calpain, the calcium-activated neutral protease, has been implicated in OGD-induced astrocytic oncosis. In a previous study, intervention with a calpain inhibitor delayed OGD-induced astrocytic oncosis, suggesting that calpain is an important mediator of oncosis [172]. Transient receptor potential melastatin 4 (TRPM4), a nonselective cation channel of oncosis, is sensitive to intracellular ATP and Ca^2+^ levels, and its expression and activity levels have been reported to be enhanced in ischemic stroke [173–175]. A recent study performed by Wei *et al*. showed that ischemia-induced activation of TRPM4 resulted in Na^+^ influx, and TRPM4 inhibition significantly attenuated ATP depletion and oncotic cell death in hypoxia-treated neurons, astrocytes and vascular endothelial cells [176]. Additionally, various ion channels and transporters that regulate ionic movement across cell membranes are involved in ischemic stroke, while the role of ion channels and transporters in oncosis still requires further investigation.

#### The interplay of regulated necrosis pathways in ischemic stroke

As stated above, ischemic stroke leads to multiple types of regulated necrosis in various CNS cells. Furthermore, mitochondria act as the core mediator of the cross-regulation between regulated necrosis in ischemic stroke. Mitochondria play a central role in ATP generation in eukaryotic cells. As a consequence, mitochondrial dysfunction caused by ischemic stroke manifests as ATP deficiency, mPTP opening, ROS generation, calcium overload and cytochrome c release [177]. CypD-mediated mPTP opening is essential to mPTP-mediated necrosis, disruption of ion homeostasis and ROS generation; therefore, it is deeply involved in necroptosis, ferroptosis, parthanatos and oncosis [178].

In ischemia–reperfusion injury, the upregulation of RIPK1 and RIPK3 results in CypD-mediated mPTP opening, Ca^2+^ influx and ROS generation, and the inhibition of CypD-mediated mPTP opening could alleviate ischemic stroke-induced necroptosis [179, 180]. ACSL4 overexpression, GPX4 inhibition and GSH downregulation in ischemic stroke lead to lipoxygenase activation, Ca^2+^ influx, mitochondrial membrane rupture and inhibition of ferroptosis, effectively attenuating mPTP opening and leading to mitochondrial dysfunction [120, 181, 182]. In addition, iron overload contributes to the overproduction of membrane peroxides, mPTP opening and necroptosis by catalyzing ROS generation and damaging the BBB in stroke [183–185]. As one of the mechanisms of parthanatos, AIF showed the ability to interact with RIPK3 in the nucleus and induce necroptosis in intracerebral hemorrhage mice, and intervention with CsA blocked the RIPK3–AIF complex by inhibiting mPTP opening [186].

Notably, ROS, which are mainly generated in mitochondria as a consequence of oxidative phosphorylation (OXPHOS), contribute to various types of regulated necrosis in ischemic stroke and act as the major mechanism of membrane lipid peroxidation, oxidative stress-triggered DNA degradation and oxidative damage to proteins [187–189]. Several studies have indicated that ROS generation leads to PARP activation, AIF release, RIPK1 autophosphorylation, RIPK3 recruitment and NLRP3 inflammasome activation in response to ischemic stroke [187, 190, 191]. In gliomas, ROS upregulation acts as an important mechanism of oncosis [192]. Meanwhile, necrosomes, the NLRP3 inflammasome and iron overload induce ROS accumulation in turn [193, 194]. Additionally, the interplay between ER stress and ROS generation via the activation of the unfolded-protein response also plays an important role in necroptosis, ferroptosis and mPTP-mediated necrosis [195–197].

Hence, as the different types of regulated necrosis are closely interrelated, inhibition of one type of regulated necrosis could effectively block the mechanisms of other regulated necrosis types. Moreover, Tian *et al*. found that the combination of emricasan and ponatinib could inhibit both apoptosis and necroptosis more effectively than inhibition of apoptosis or necroptosis alone in ischemic stroke rats [198]. Hence, compounds that target multiple types of regulated necrosis may be a potential treatment for ischemic stroke.

## Conclusions

Due to its high morbidity, mortality and disability rates, ischemic stroke has become an intractable issue for public health, especially in China. In addition to apoptosis, necrosis and autophagy, which are traditionally the three major types of cell death, growing evidence has indicated that several types of regulated necrosis, including necroptosis, pyroptosis, ferroptosis, parthanatos, mPTP-mediated necrosis and oncosis, contribute to CNS cell death in ischemic stroke. Interestingly, a number of studies have found that ischemic stroke-induced regulated necrosis is interconnected and closely related to the functions of organelles such as mitochondria and the ER. Notably, various traditional Chinese medicines have been reported to inhibit ischemic stroke-induced regulated necrosis. For example, kaempferol alleviates OGD/R injury by inhibiting ferroptosis and ROS generation. To alleviate ischemic stroke, inhibiting multiple types of regulated necroptosis has been found to be more effective than blocking a single pathway. Hence, investigating the mechanisms of regulated necrosis and the interplay among various regulated necrosis types could shed light on a novel direction to treat ischemic stroke injury. However, many unknown factors need to be uncovered before these strategies can be applied in the clinical treatment of ischemic stroke.

## Funding

This work was supported by the National Key Research and Development Program of China (No. 2020YFC2008304), Natural Science Foundation of Henan Province (No. 222300420350), National Natural Science Foundation of China (No. 82204389), Medical Science and Technique Foundation of Henan Province (No. LHGJ20200310, SBGJ202102145) and Foundation of Henan Educational Committee (No. 21A310027, No. 21A350014), National Natural Science Young Scientists Foundation of China (No. 82200796), China Postdoctoral Science Foundation (No. 2022M722901).

## Data availability

All data generated or analyzed in this study are available from the corresponding author on reasonable request.

## Authors’ contributions

KR, JP, YG, YJ, QF and YY conceptualized and wrote the manuscript and created the figures. KR, HX, YX and QF contributed to writing the manuscript. KR, YY, QF and JY reviewed and modified the manuscript. All authors approved the final version of the manuscript.

## Conflicts of interests

All the authors in this study declare that there are no competing interests.
